# Supplementary material for: Fabrication of Polypyrrole Hollow Nanospheres by Hard-Template Method for Supercapacitor Electrode Material
Source: Molecules. 2024 May 15;29(10):2331. doi: 10.3390/molecules29102331 (PMC11124273; doi:10.3390/molecules29102331)
Supplement: Supplementary file 1 [file molecules-29-02331-s001.zip › molecules-3004105-supplementary.pdf]

Supporting Information

Fabrication of Polypyrrole Hollow Nanospheres by Hard-Template Method for Supercapacitor Electrode Material

Renzhou Hong, Xijun Zhao, Rongyu Lu, Meng You, Xiaofang Chen * and Xiaoming Yang *

State and Local Joint Engineering Laboratory for Novel Functional Polymeric Materials, Jiangsu Engineering Laboratory of Novel Functional Polymeric Materials, Suzhou Key Laboratory of Macromolecular Design and Precision Synthesis, Department of Polymer Science and Engineering, College of Chemistry, Chemical Engineering and Materials Science, Soochow University, Suzhou 215123, China; 20214209184@stu.suda.edu.cn (R.H.); 20214209280@stu.suda.edu.cn (X.Z.); 20224209228@stu.suda.edu.cn (R.L.); 20234209022@stu.suda.edu.cn (M.Y.)

* Correspondence: xfchen75@suda.edu.cn (X.C.); yangxiaoming@suda.edu.cn (X.Y.)

The supporting information includes:

Experimental section

Chromatogram report

Header

Title		Data acquisition date and time	2023/11/27 20:52:02
Sample name	PS	Calculation date and time	2023/11/29 21:22:22
Database name	2023-11-27.chd	Acquisition time [min]	0.000 - 15.000
Data name	RSLT0025	Sampling interval [msec]	100
Method name	THF-20230911-PS	Cup number	13
Channel	RI	Calculation type	Molecular Weight

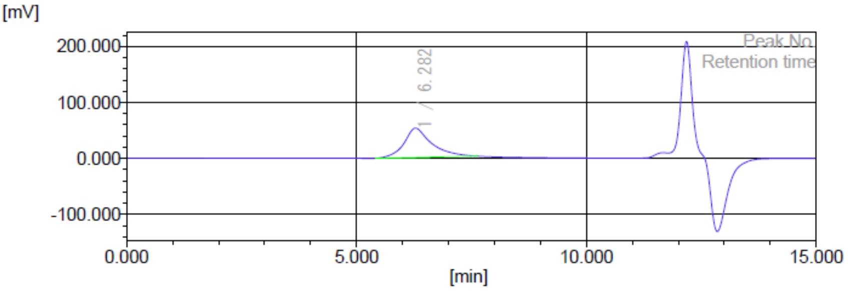

Result of molecular weight calculation (RI)

Peak 1 Base Peak

	[min]	[mV]	[mol]		
Peak start	5.420	0.235	15,681,153	Mn	115,761
Peak top	6.282	54.180	194,905	Mw	293,244
Peak end	7.623	4.007	19,569	Mz	1,327,279
				Mz+1	4,359,408
				Mv	293,244
Height [mV]			52.470	Mp	168,014
Area [mV*sec]			2201.594	Mz/Mw	4.526
Area% [%]			100.000	Mw/Mn	2.533
[eta]			293243.74398	Mz+1/Mw	14.866

Result of molecular weight calculation (RI)

Total

	[min]	[mV]	[mol]		
Peak start	5.420	0.235	15,681,153	Mn	115,761
Peak top	6.282	54.180	194,905	Mw	293,244
Peak end	7.623	4.007	19,569	Mz	1,327,279
				Mz+1	4,359,408
				Mv	293,244
Height [mV]			52.470	Mp	168,014
Area [mV*sec]			2201.594	Mz/Mw	4.526
Area% [%]			100.000	Mw/Mn	2.533
[eta]			293243.74398	Mz+1/Mw	14.866

Figure S1. Gel Permeation Chromatography (GPC) trace of PS core.

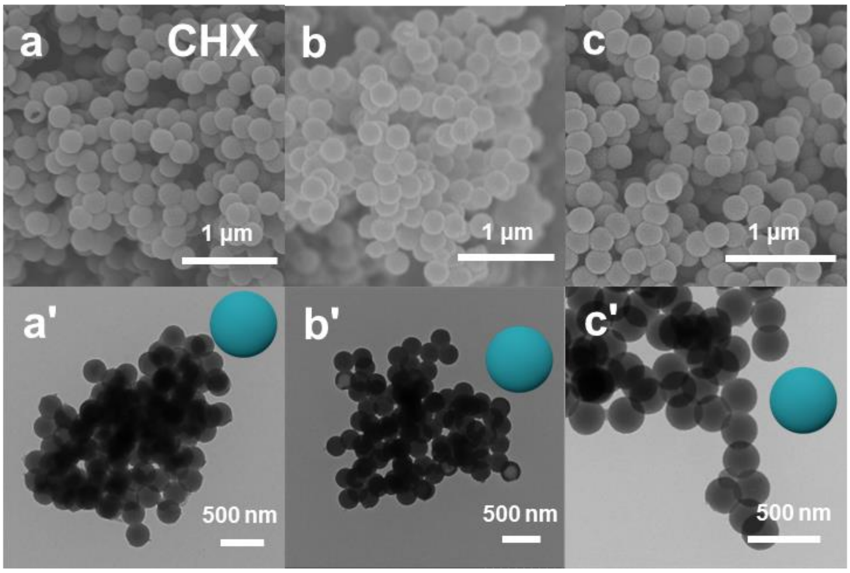

Figure S2. SEM and corresponding TEM images of (a, a′) CHX PS@PPy20, (b, b′) CHX PS@PPy30, and (c, c′) CHX PS@PPy40.

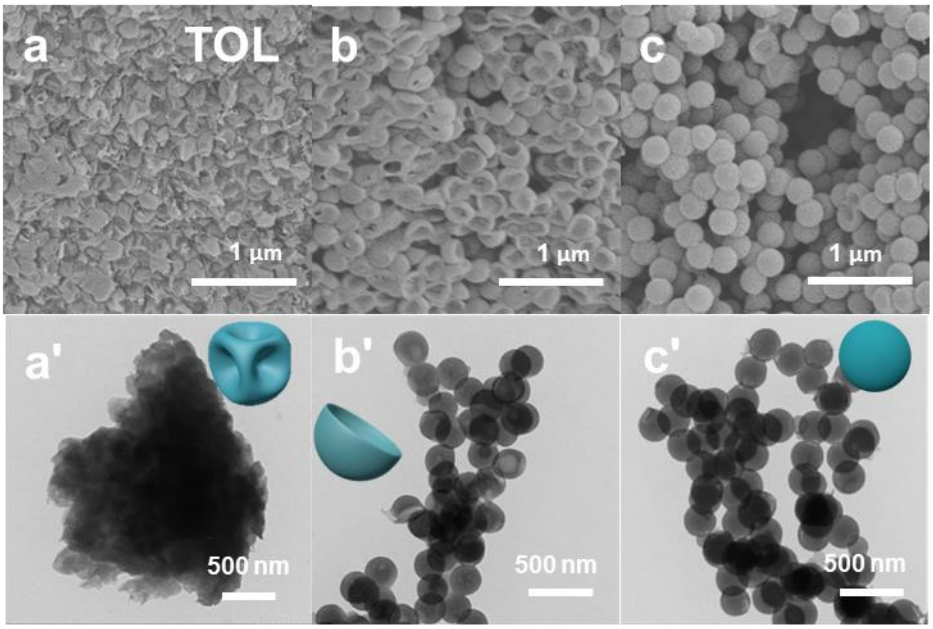

Figure S3. SEM and corresponding TEM images of (a, a’) TOL PS@PPy20, (b, b’) TOL PS@PPy30, and (c, c’) TOL PS@PPy40.

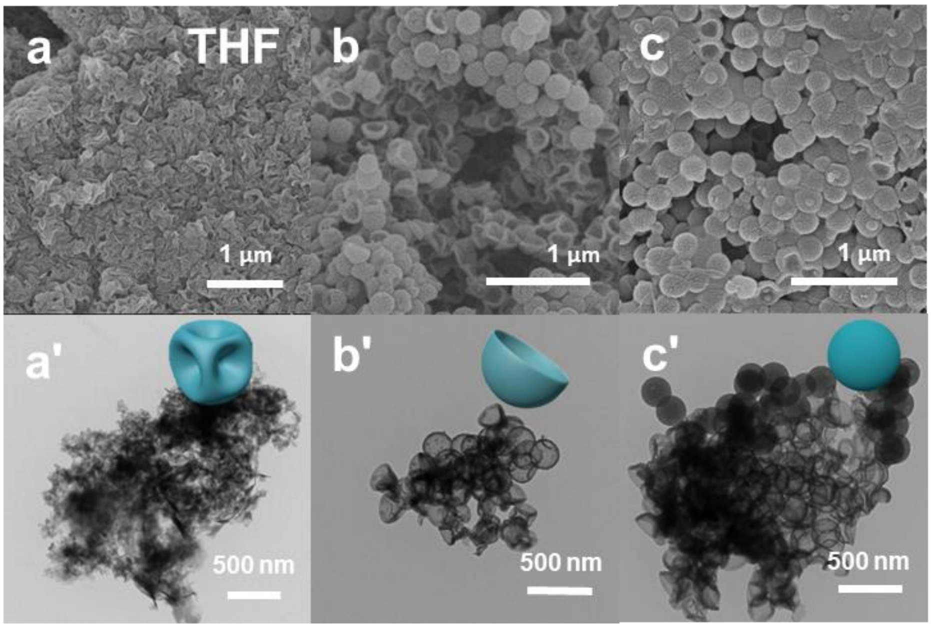

Figure S4. SEM and corresponding TEM images of (a, a’) THF PS@PPy20, (b, b’) THF PS@PPy30, and c, c’) THF PS@PPy40.

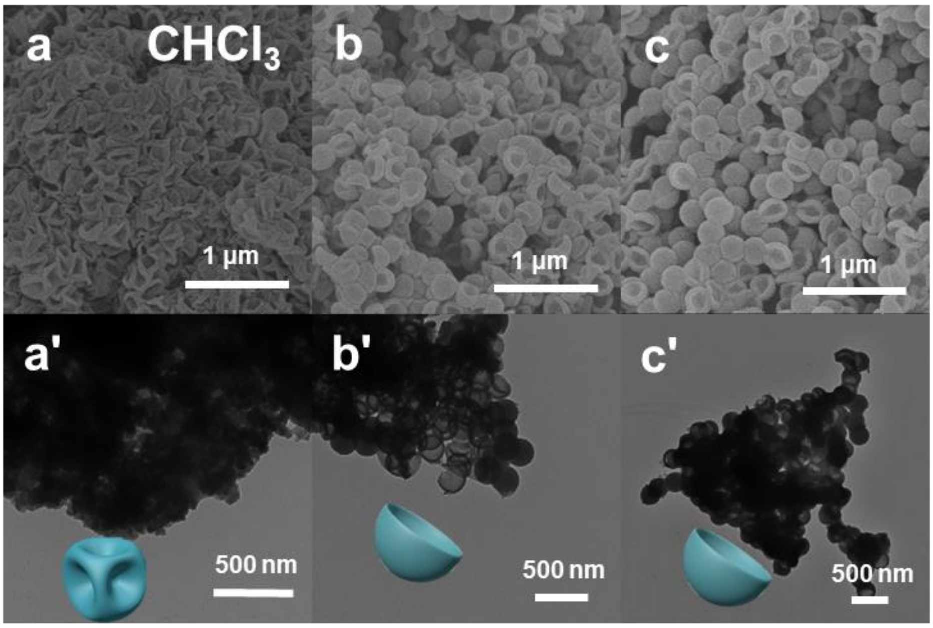

Figure S5. SEM and corresponding TEM images of (a, a′) $CHCl_3$ PS@PPy20, (b, b′) $CHCl_3$ PS@PPy30, and (c, c′) $CHCl_3$ PS@PPy40.

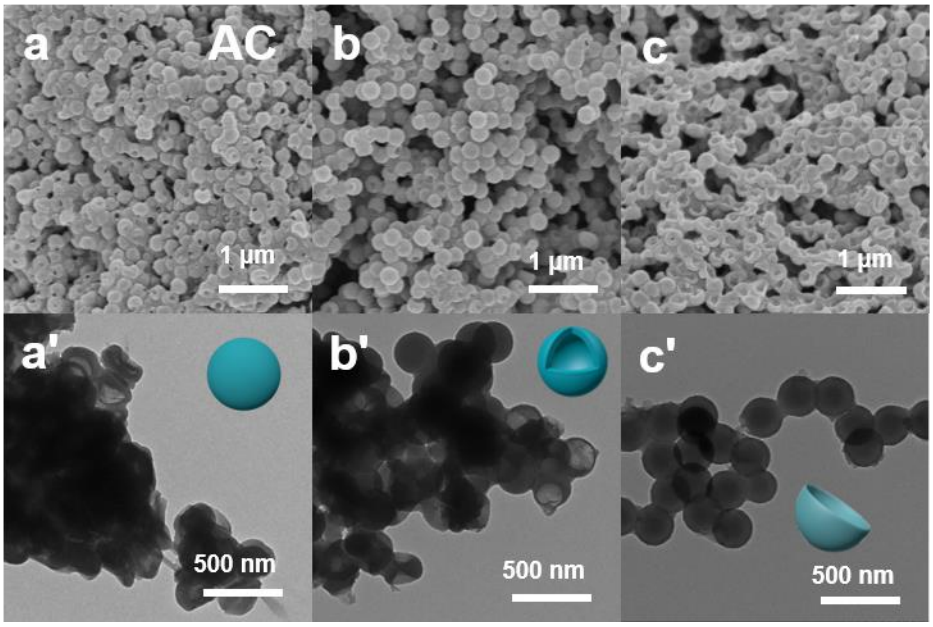

Figure S6. SEM and corresponding TEM images of (a,a′) AC PS@PPy20, (b,b′) AC PS@PPy30, and (c,c′) AC PS@PPy40.

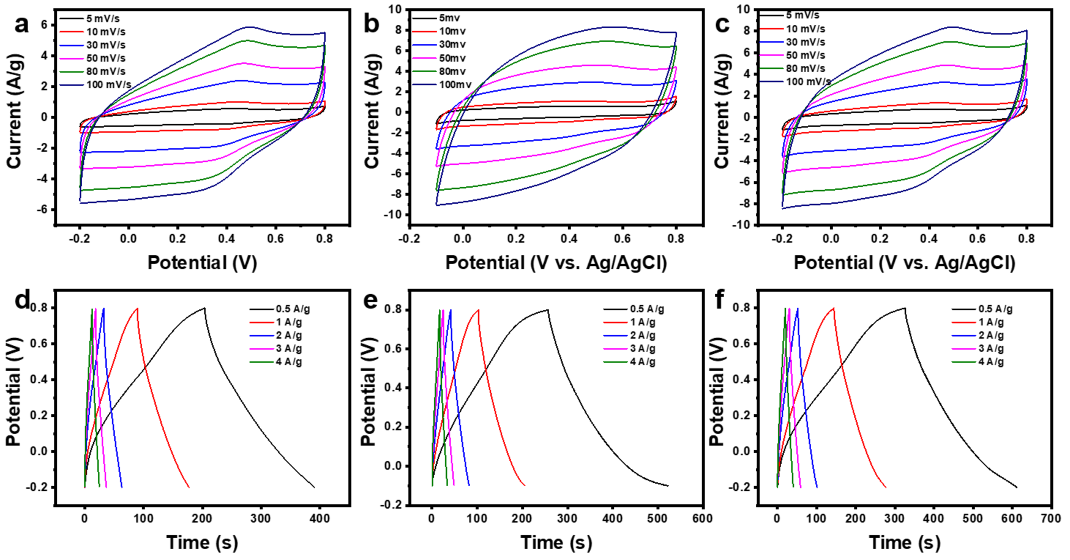

Figure S7. CV curves of (a) CHX PS@PPy20, (b) CHX PS@PPy30 and (c) CHX PS@PPy40 at different scan rates of 5 to 100 mV s^{-1} curve; GCD curves of (d)CHX PS@PPy20, (e) CHX PS@PPy30 and (f) CHX PS@PPy40 under different current density of 0.5 to 4 A g^{-1}.

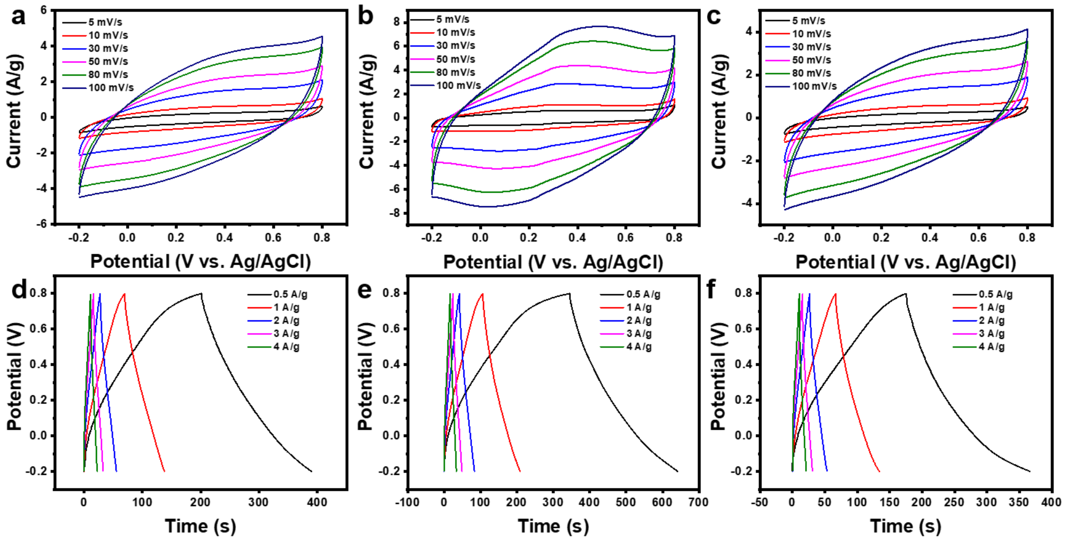

Figure S8. CV curves of (a)TOL PS@PPy20, (b) TOL PS@PPy30 and (c) TOL PS@PPy40 at different scan rates of 5 to 100 mV s^{-1} curve; GCD curves of (d)TOL PS@PPy20, (e) TOL PS@PPy30 and (f) TOL PS@PPy40 under different current density of 0.5 to 4 A g^{-1}.

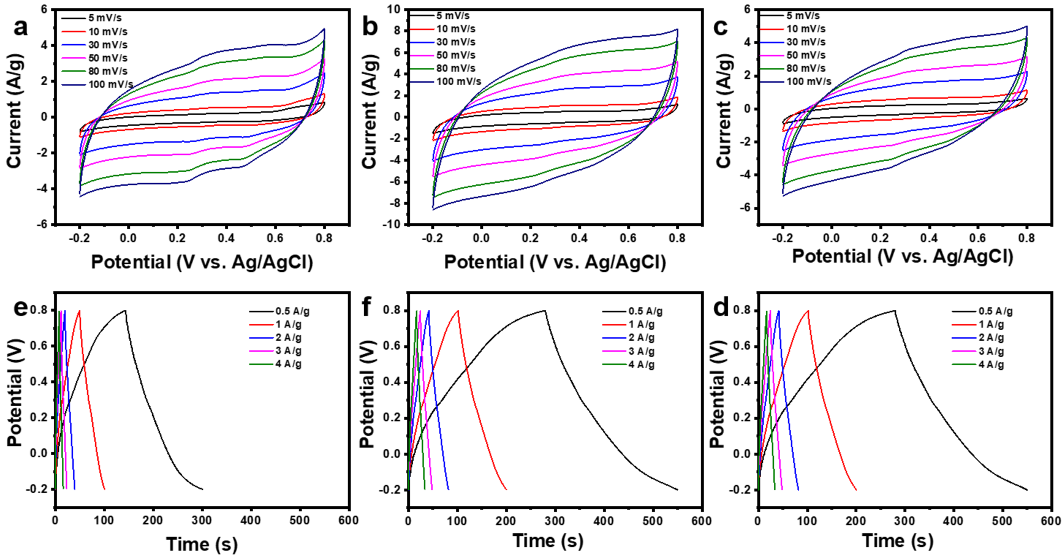

Figure S9. CV curves of (a)THF PS@PPy20, (b) THF PS@PPy30 and (c) THF PS@PPy40 at different scan rates of 5 to 100 mV s^{-1} curve; GCD curves of (d)THF PS@PPy20, (e) THF PS@PPy30 and (f) THF PS@PPy40 under different current density of 0.5 to 4 A g^{-1}.

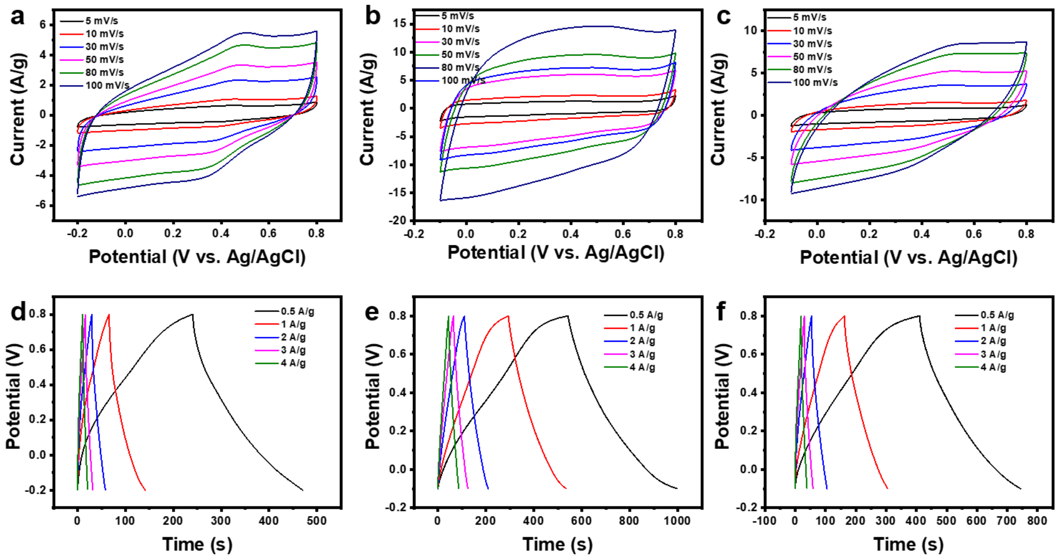

Figure S10. CV curves of (a) $CHCl_3$ PS@PPy20, (b) $CHCl_3$ PS@PPy30 and (c) $CHCl_3$ PS@PPy40 at different scan rates of 5 to 100 mV s^{-1} curve; GCD curves of (d) $CHCl_3$ PS@PPy20, (e) $CHCl_3$ PS@PPy30 and (f) $CHCl_3$ PS@PPy40 under different current density of 0.5 to 4 A g^{-1}.

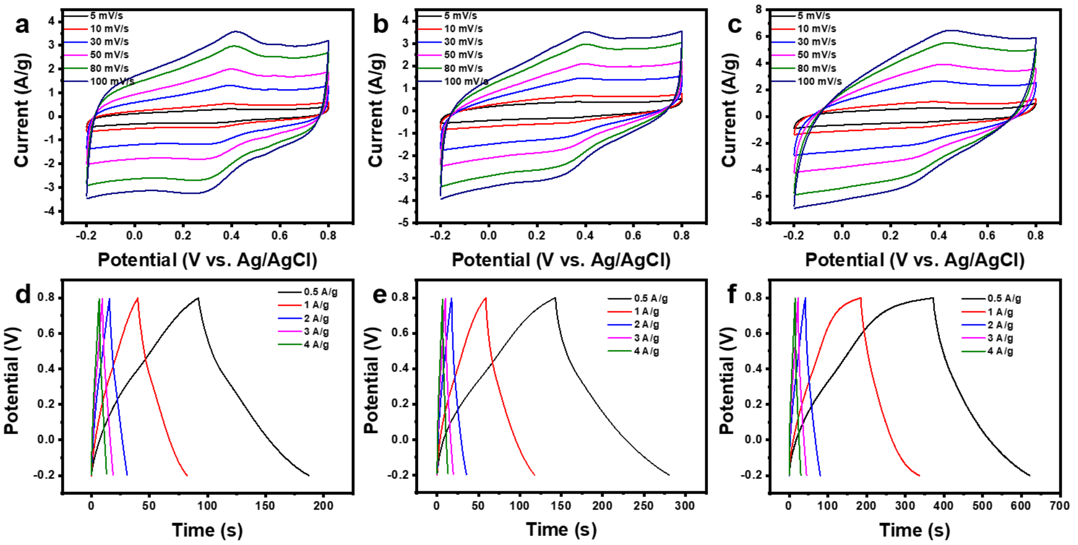

Figure 11. CV curves of (a)AC PS@PPy20, (b)AC PS@PPy30 and (c) AC PS@PPy40 at different scan rates of 5 to 100 mV s^{-1} curve; GCD curves of (d)AC PS@PPy20, (e) AC PS@PPy30 and (f) AC PS@PPy40 under different current density of 0.5 to 4 A g^{-1}.

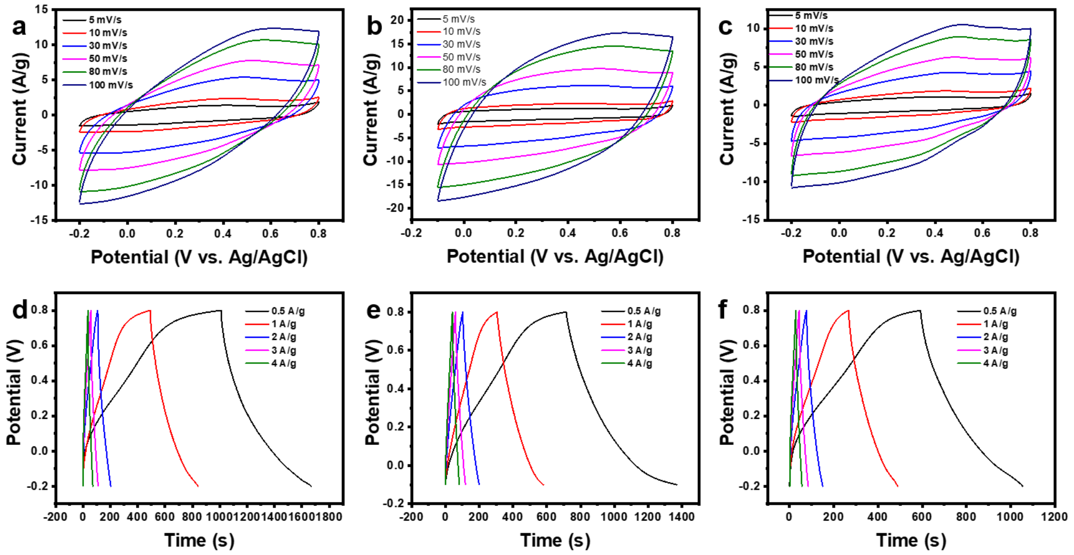

Figure S12. CV curves of (a)DMF PS@PPy20, (b) DMF PS@PPy30 and (c) DMF PS@PPy40 at different scan rates of 5 to 100 mV s^{-1} curve; GCD curves of (d)DMF PS@PPy20, (e) DMF PS@PPy30 and (f) DMF PS@PPy40 under different current density of 0.5 to 4 A g^{-1}.

Table S1 Specific capacitances of organic solvents etching PS@PPy

Capacitance (F/g)	0.5 A/g	1 A/g	2 A/g	3 A/g	4 A/g
PS@PPy20	49	44	40	37	36
PS@PPy30	70	65	47	38	31
PS@PPy40	100	82	70	62	58
CHX PS@PPy20	93	88	62	54	48
CHX PS@PPy30	147	114	90	80	74
CHX PS@PPy40	143	132	98	87	79
TOL PS@PPy20	95	68	55	49	45
TOL PS@PPy30	148	102	83	73	67
TOL PS@PPy40	96	67	53	46	42
THF PS@PPy20	78	50	40	35	32
THF PS@PPy30	135	98	80	72	66
THF PS@PPy40	111	84	70	63	58
$CHCl_3$ PS@PPy20	115	76	56	47	41
$CHCl_3$ PS@PPy30	270	268	219	199	188
$CHCl_3$ PS@PPy40	185	158	113	96	84
AC PS@PPy20	48	42	30	27	26
AC PS@PPy30	69	59	36	29	26
AC PS@PPy40	124	110	76	66	59
DMF PS@PPy20	360	350	190	160	140
DMF PS@PPy30	362	303	217	194	181
DMF PS@PPy40	231	221	147	123	110

Table S2 EIS characteristics of DMF PS@PPy

	R_s (Ω)	C_1(mF)	R_{ct1}(Ω)	C_2(F)	W (Ω)
DMF PS@PPy20	1.13	5.3	0.01	0.32	0.27
DMF PS@PPy30	1.38	0.11	0.27	0.24	0.13
DMF PS@PPy40	1.54	0.11	0.45	0.12	0.01

Table S3 Volumetric capacitance of organic solvents etching PS@PPy

Capacitance (F/cm^3)	0.5 A/g	1 A/g	2 A/g	3 A/g	4 A/g
PS@PPy20	20	18	16	15	14
PS@PPy30	11	10	8	6	5
PS@PPy40	13	11	10	8	7
CHX PS@PPy20	34	32	23	20	18
CHX PS@PPy30	23	18	14	13	12
CHX PS@PPy40	28	26	20	17	16
TOL PS@PPy20	53	38	31	27	25
TOL PS@PPy30	34	23	19	17	15
TOL PS@PPy40	17	12	10	8	7
THF PS@PPy20	57	37	29	27	23

THF PS@PPy30	15	11	9	8	7
THF PS@PPy40	18	13	11	10	9
CHCl3 PS@PPy20	57	38	28	23	20
CHCl3 PS@PPy30	20	21	17	16	15
CHCl3 PS@PPy40	18	16	11	10	8
AC PS@PPy20	7	6	5	4	4
AC PS@PPy30	13	11	7	6	5
AC PS@PPy40	17	15	11	9	8
DMF PS@PPy20	159	154	83	70	62
DMF PS@PPy30	69	57	41	37	34
DMF PS@PPy40	25	24	16	13	12

Table S4 EIS characteristics of DMF PS@PPy20 based flexible supercapacitors

	R_s (Ω)	C_1(mF)	R_{ct1}(Ω)	C_2(F)	W (Ω)
DMF PS@PPy20	5.46	0.06	0.6	0.009	0.04
